# Supplementary material for: Rapid movement and transcriptional re‐localization of human cohesin on DNA
Source: EMBO J. 2016 Oct 31;35(24):2671–85. doi: 10.15252/embj.201695402 (PMC5167347; doi:10.15252/embj.201695402)
Supplement: Supplementary file 1 — Appendix [file EMBJ-35-2671-s001.pdf]

Rapid movement and transcriptional re-localization of human cohesin on DNA

Davidson et al.

Appendix

Table of contents:

Appendix Figures S1 – S7 Pages 2 – 8

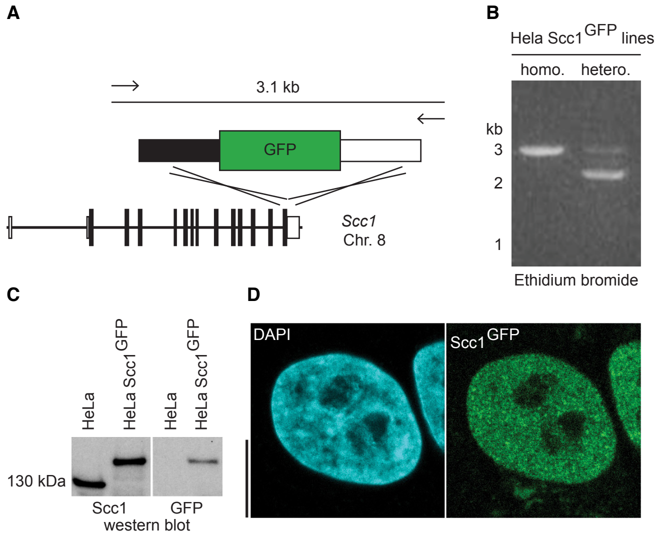

Appendix Figure S1. HeLa Kyoto Scc1^{GFP} cell line generation.

A Schematic representation of the HeLa Kyoto Scc1 locus. The GFP cassette and flanking homology arms was introduced at exon 14 before the Scc1 stop codon. Insertion was verified by PCR using primers external to the homology arms (primer annealing sites labelled by arrows).

B PCR products generated with primers described in (A) using genomic DNA extracted from a homozygous (lane 1) and a heterozygous (lane 2) HeLa Kyoto Scc1GFP cell line. The single PCR product generated from the homozygous cell line DNA confirmed homozygous insertion of the GFP cassette. The two PCR products generated from the heterozygous cell line DNA represent the region surrounding the stop codon with and without the GFP cassette.

C Chromatin fractions isolated from HeLa Kyoto and homozygous HeLa Kyoto Scc1GFP cells were separated by SDS–PAGE. Scc1 and GFP were detected by immunoblotting.

D Confocal microscopy images of the endogenous GFP signal from a homozygous HeLa Kyoto Scc1^{GFP} cell co-stained with DAPI. Scale bar, 10 µm.

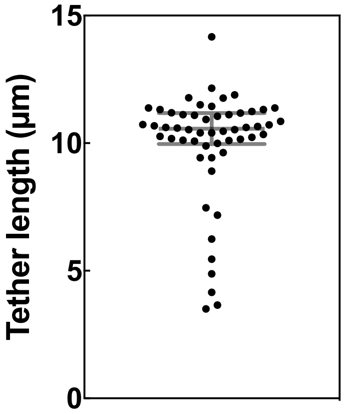

Appendix Figure S2. Tether length of doubly tethered λ-phage DNA in the absence of buffer flow. Median and interquartile range are shown; n = 54.

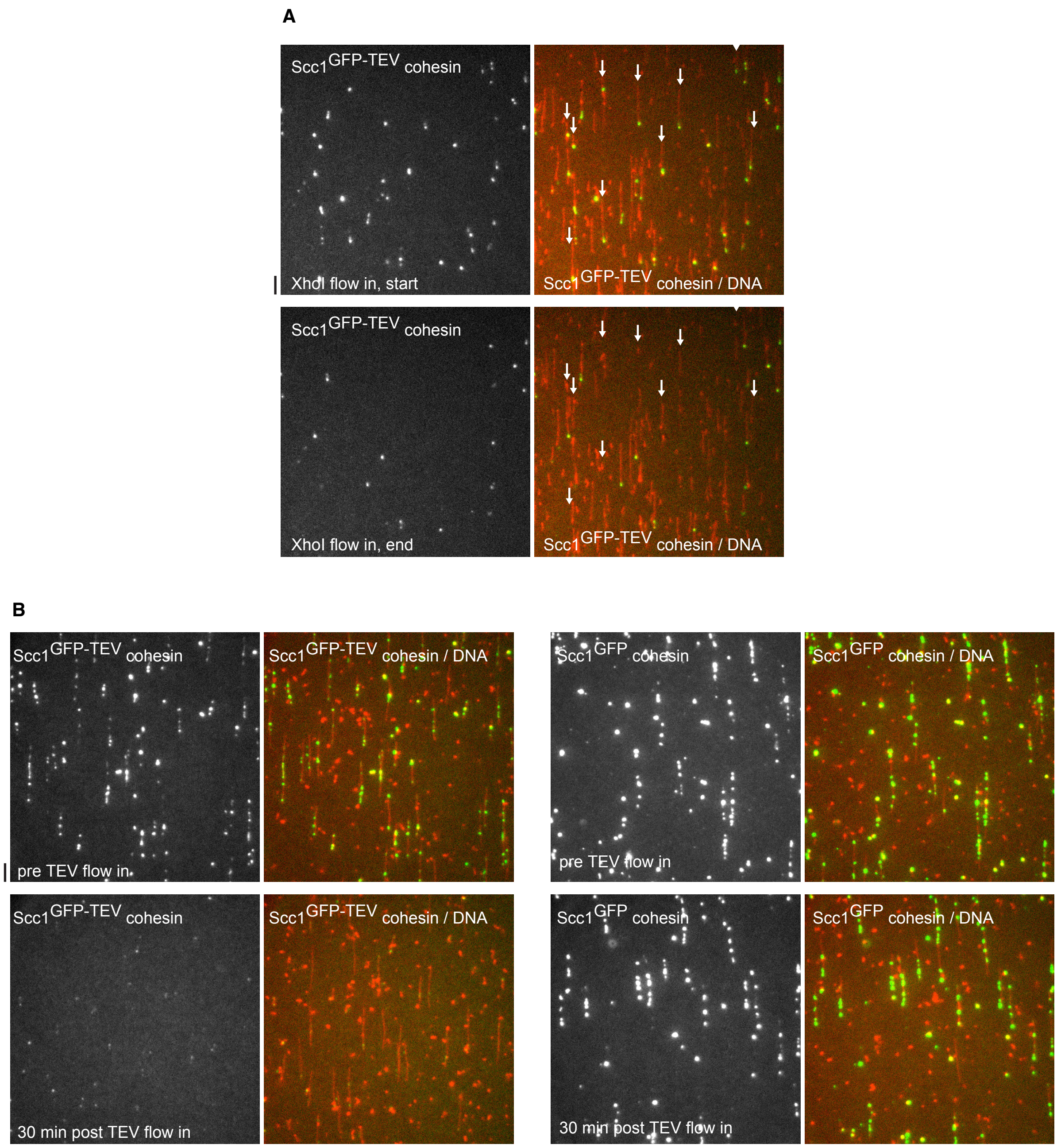

Appendix Figure S3. Cohesin is released following DNA or cohesin cleavage.

A Representative field of view showing Scc1 $^{GFP\text{-}TEV}$-cohesin bound to λ-DNA after 750 mM NaCl wash + Sytox Orange. Upper panels: before XhoI flow in; lower panels: same field of view after XhoI flow in. Arrows denote all DNA molecules cleaved by XhoI.

B Representative fields of view showing Scc1 $^{GFP\text{-}TEV}$-cohesin (left panels) and Scc1 ^{GFP} cohesin (right panels) bound to λ-DNA after 750 mM NaCl wash + Sytox Orange. Upper panels: before TEV protease flow in; lower panels: 30 min after TEV protease flow in.

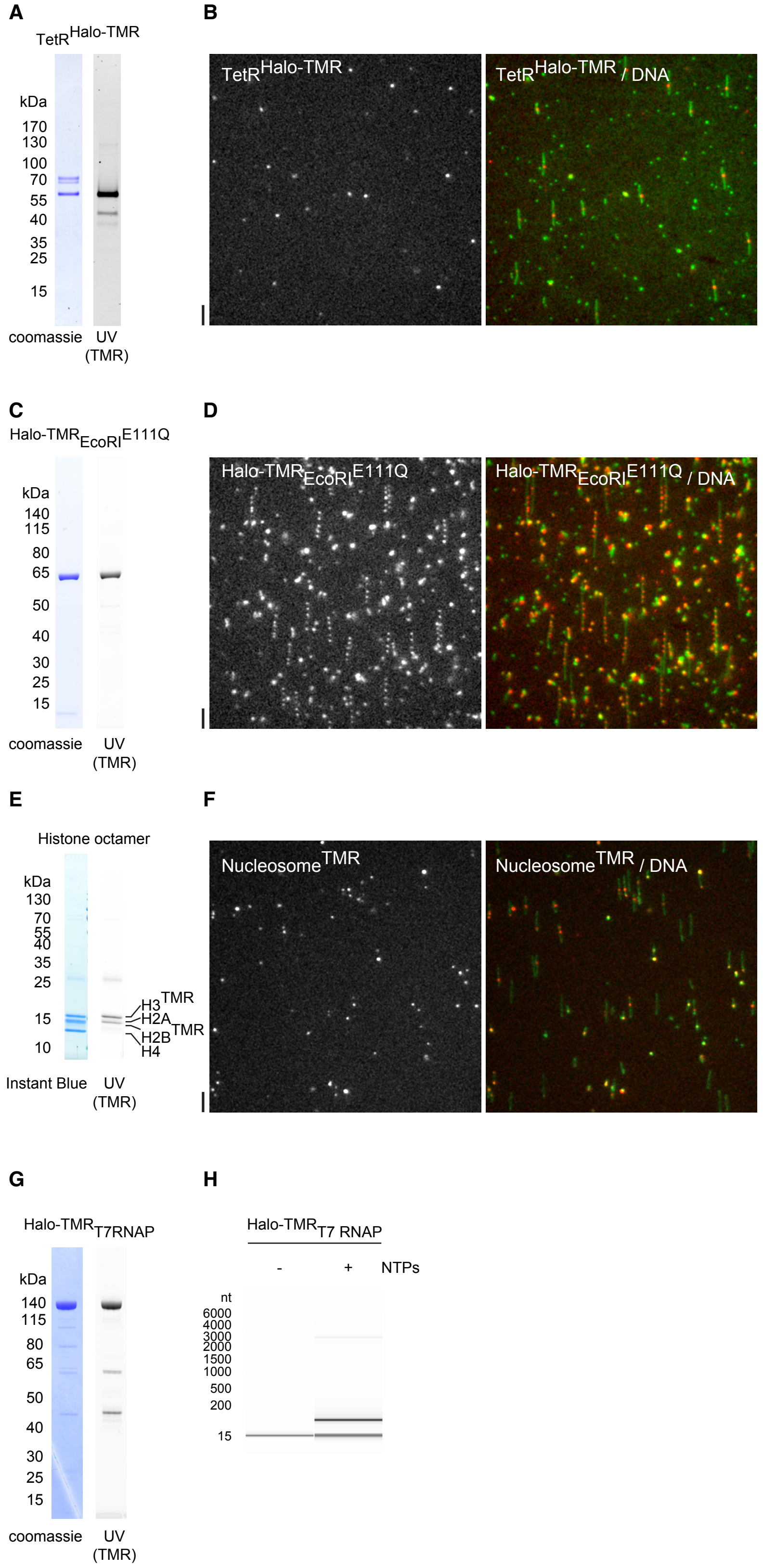

Appendix Figure S4. Characterisation of TetR$^{Halo\text{-}TMR}$, $^{Halo\text{-}TMR}$EcoRI^{E111Q}, TMR-labelled nucleosomes and ^{Halo}TMRT7 RNAP.

A, C, E, G Coomassie / Instant Blue staining of purified recombinant TetR$^{Halo\text{-}TMR}$, $^{Halo\text{-}TMR}$EcoRI^{E111Q}, H2A/H2B^{TMR}/H3^{TMR}/H4 histone octamers and $^{Halo\text{-}TMR}$T7 RNAP after SDS–PAGE. TMR was visualized by UV excitation.

B, D, F Representative fields of view showing TetR$^{Halo\text{-}TMR}$ bound to pPlat-TetO DNA (TetO at position 10123 bp), $^{Halo\text{-}TMR}$EcoRI^{E111Q} bound to λ-DNA and TMR-labelled nucleosomes bound to pPlat-601 DNA (601 at position 10123 bp) after 150 mM NaCl wash + Sytox Green. The λ-genome contains five EcoRI recognition sequences (21,226; 26,104; 31,747; 32,407; 41,808 bp).

H In vitro transcription of a 119 nt RNA using Halo-TMRT7 RNAP. Purified reaction products were separated using capillary electrophoresis.

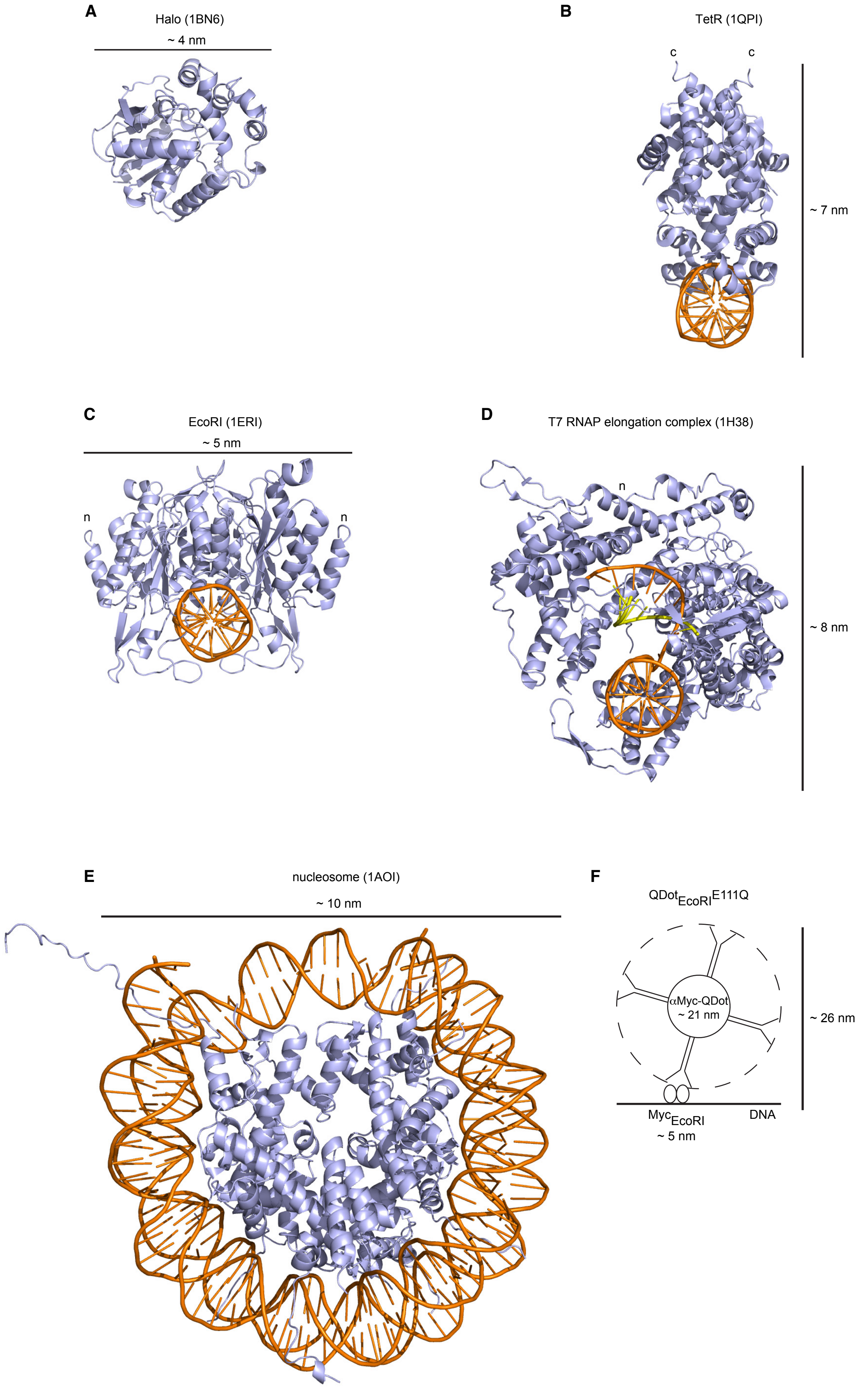

Appendix Figure S5. Dimensions of HaloTag, TetR, EcoRI, T7 RNAP, nucleosomes and ^{QDot}EcoRI^{E111Q}.

A-E Estimation of roadblock protein dimensions from crystal structures. HaloTag was fused to the carboxy terminus of TetR (c) and to the amino terminus of EcoRI^{E111Q} and T7 RNAP (n). TetR and EcoRI^{E111Q} bind to DNA as homodimers. PDB accession codes are shown in parentheses.

F Approximate dimensions of ^{QDot}EcoRI^{E111Q} roadblock.

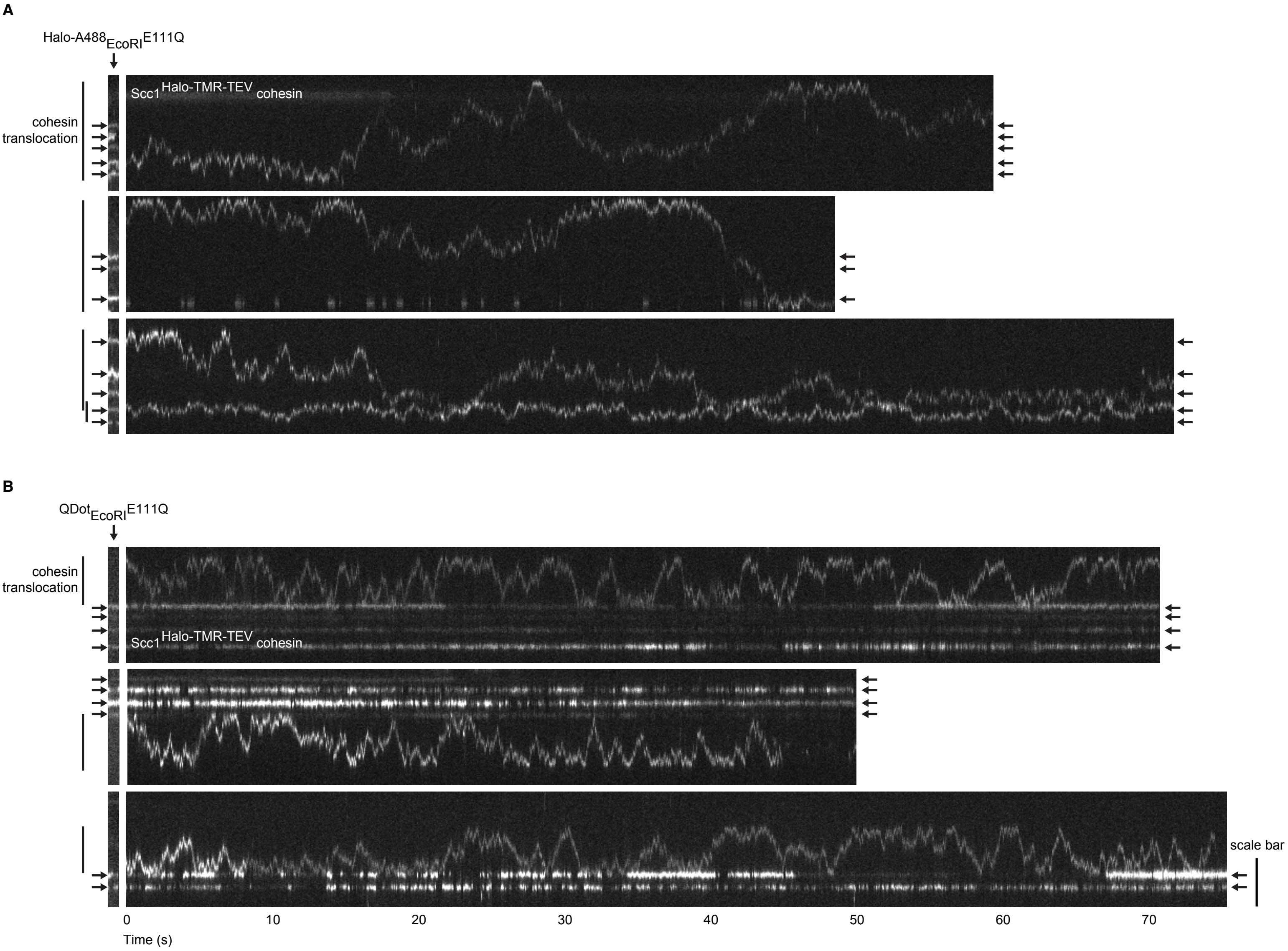

Appendix Figure S6. Cohesin bypasses DNA-bound $^{\text{Halo-A488}}$EcoRI$^{\text{E111Q}}$ but not ^{QDot}EcoRI$^{\text{E111Q}}$.

A High temporal resolution kymographs of salt-resistant Scc1$^{\text{Halo-TMR-TEV}}$-cohesin diffusing past DNA-bound $^{\text{Halo-A488}}$EcoRI$^{\text{E111Q}}$. $^{\text{Halo-A488}}$EcoRI$^{\text{E111Q}}$ and cohesin were illuminated sequentially; arrows highlight positions of $^{\text{Halo-A488}}$EcoRI$^{\text{E111Q}}$.

B High temporal resolution kymographs of salt-resistant Scc1$^{\text{Halo-TMR-TEV}}$-cohesin failing to bypass DNA-bound ^{QDot}EcoRI$^{\text{E111Q}}$. ^{QDot}EcoRI$^{\text{E111Q}}$ and cohesin were illuminated sequentially; ^{QDot}EcoRI$^{\text{E111Q}}$ was also detectable in cohesin channel. Arrows highlight positions of ^{QDot}EcoRI$^{\text{E111Q}}$.

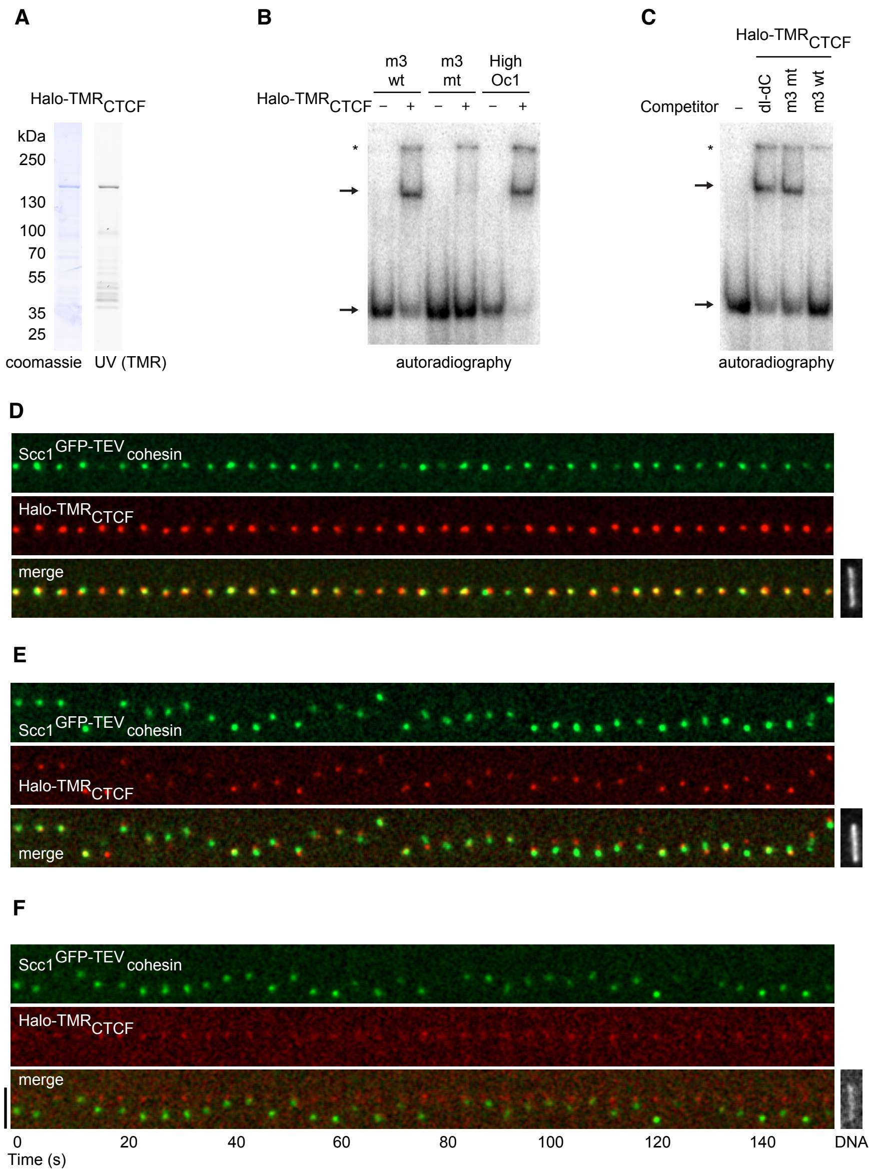

Appendix Figure S7. Characterisation of $^{\text{Halo-TMR}}$CTCF.

A Coomassie staining of purified recombinant $^{\text{Halo-TMR}}$CTCF after SDS–PAGE. TMR was visualized by UV excitation.

B-C Electrophoretic mobility shift assays. (B) Radiolabelled probes containing a wild type CTCF binding site from the H19/IGF2 locus (m3 wt), a mutated version (m3 mt) or a high affinity CTCF binding site (HighOc1) were incubated with $^{\text{Halo-TMR}}$CTCF and excess poly(dI-dC).poly(dI-dC) non-specific competitor. Free and CTCF-bound probe (arrows) were separated by PAGE and detected using a phosphorimager. Asterisk denotes material trapped in the gel wells. (C) Radiolabelled m3 wt probe was incubated with $^{\text{Halo-TMR}}$CTCF and excess unlabeled poly(dI-dC).poly(dI-dC), m3 mt or m3 wt competitor DNA and processed as in (B).

D-E Kymographs of pPlat-4xCTCF DNA-bound $^{\text{Halo-TMR}}$CTCF and Scc1$^{\text{GFP-TEV}}$-cohesin. DNA was post-stained with Sytox Green. Of 88 instances in a representative field of view in which cohesin and CTCF occupied the same doubly-tethered DNA molecule, cohesin and CTCF colocalised in 72 cases, most often without moving (D) but in a few instances while translocating together along DNA (E).

F Kymograph of salt-resistant Scc1$^{\text{GFP-TEV}}$-cohesin failing to bypass $^{\text{Halo-TMR}}$CTCF bound at a single CTCF binding site (pPlat-1xCTCF). DNA was post-stained with Sytox Green.
