# Supplementary material for: Rapid movement and transcriptional re‐localization of human cohesin on DNA
Source: EMBO J. 2016 Oct 31;35(24):2671–85. doi: 10.15252/embj.201695402 (PMC5167347; doi:10.15252/embj.201695402)
Supplement: Supplementary file 2 — Expanded View Figures PDF [file EMBJ-35-2671-s002.pdf]

Expanded View Figures

Figure EV1. Characterization of recombinant human tetrameric cohesin complexes.

A Cohesin loading assay. Scc1$^{Halo\text{-}TMR\text{-}TEV}$-cohesin tetramer was incubated with nicked circular plasmid DNA, immunoprecipitated with anti-Scc1 antibodies, and washed with high-salt buffer. DNA was eluted using proteinase K, separated by agarose gel electrophoresis, and stained with GelRed DNA stain. Input DNA = 10%. Mean ± SEM are shown.

B Silver staining of purified recombinant human Scc1^{GFP}, Scc1$^{GFP\text{-}TEV}$ and Scc1$^{Halo\text{-}TMR\text{-}TEV}$-cohesin tetramers after SDS–polyacrylamide gel electrophoresis (PAGE). TMR was visualized by UV excitation.

C *Xenopus* sperm chromatin, purified human cohesin, and interphase *Xenopus* egg extract were incubated for 75 min. TEV protease was then added for 15 min to cleave Scc1^{TEV}. Chromatin-bound material was analyzed by immunoblotting. Human and *Xenopus* Scc1 can be distinguished by the GFP/Halo-induced mobility shift.

D Kymographs of Scc1$^{GFP\text{-}TEV}$-cohesin binding to singly or doubly tethered bacteriophage λ genomic DNA in cohesin binding buffer + Sytox Orange. Doubly tethered DNA molecules were extended in the presence and absence of buffer flow, whereas singly tethered DNA molecules were only stretched under flow. The diffusion of Scc1$^{GFP\text{-}TEV}$-cohesin on doubly tethered DNA was minimal in low-salt buffer. Scc1$^{GFP\text{-}TEV}$-cohesin rapidly compacted singly tethered DNA.

E Kymograph of Scc1$^{GFP\text{-}TEV}$-cohesin bound to λ-DNA during buffer exchange from cohesin binding buffer to 750 mM NaCl buffer.

F Kymograph of Scc1$^{Halo\text{-}TMR\text{-}TEV}$-cohesin bound to doubly tethered λ-DNA in cohesin binding buffer and washed with 750 mM NaCl buffer + Sytox Green. Note the existence of bright and dim cohesin complexes on DNA. The DNA broke spontaneously at 188 s, releasing translocating cohesin complexes.

G Representative field of view showing Scc1$^{Halo\text{-}TMR\text{-}TEV}$-cohesin bound to λ-DNA in cohesin binding buffer.

H Representative field of view showing Scc1$^{Halo\text{-}TMR\text{-}TEV}$-cohesin bound to λ-DNA after 750 mM NaCl wash. Upper panels: flow off; lower panels: flow on. Scc1$^{Halo\text{-}TMR\text{-}TEV}$-cohesin was pushed to the ends of doubly tethered DNA molecules by buffer flow.

Data information: Flow in from top and scale bar = 5 μm in all kymographs.
Source data are available online for this figure.

 © 2016 The Authors

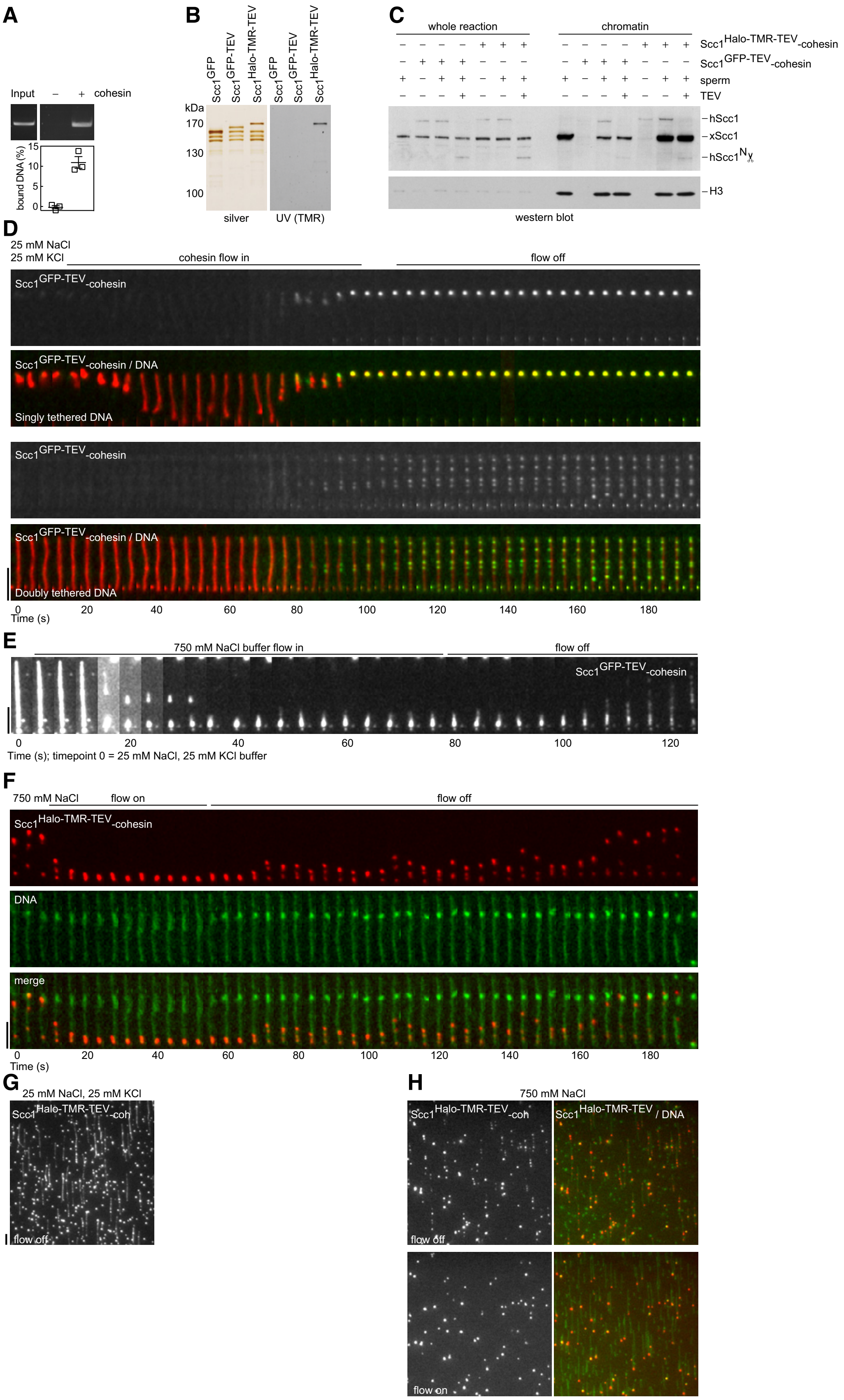
A
Input
–
+
cohesin
bound DNA (%)
B
kDa
170
130
100
silver
UV (TMR)
C
whole reaction
chromatin
Scc1Halo-TMR-TEV-cohesin
Scc1GFP-TEV-cohesin
sperm
TEV
hScc1
xScc1
hScc1N
H3
western blot
D
25 mM NaCl
25 mM KCl
cohesin flow in
flow off
Scc1GFP-TEV-cohesin
Scc1GFP-TEV-cohesin / DNA
Singly tethered DNA
Doubly tethered DNA
Time (s)
E
750 mM NaCl buffer flow in
flow off
Time (s); timepoint 0 = 25 mM NaCl, 25 mM KCl buffer
F
750 mM NaCl
flow on
flow off
Scc1Halo-TMR-TEV-cohesin
DNA
merge
Time (s)
G
25 mM NaCl, 25 mM KCl
Scc1Halo-TMR-TEV-coh
flow off
H
750 mM NaCl
Scc1Halo-TMR-TEV-coh
Scc1Halo-TMR-TEV / DNA
flow off
flow on

Figure EV1.

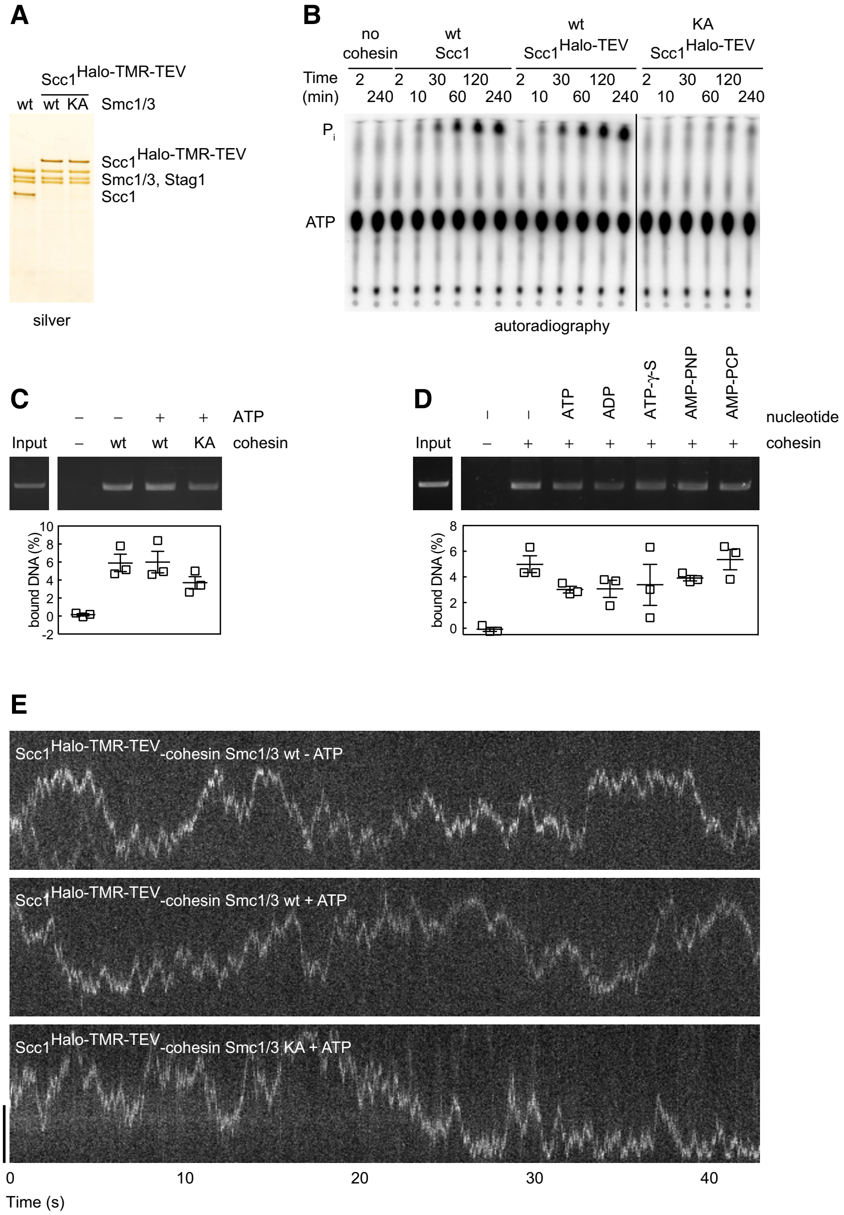

Figure EV2. ATP is not required for the salt-resistant binding of cohesin to DNA and does not affect the diffusion coefficient of cohesin on DNA.

A Silver stained SDS–polyacrylamide gel of cohesin complexes used in (B–F).

B Thin-layer chromatography of [γ-^{32}P]-ATP hydrolysis following incubation with cohesin.

C, D Smc1/3 wild-type or K38A ATP binding-deficient "KA" Scc1$^{\text{Halo-TMR-TEV}}$-cohesin was incubated with nicked circular plasmid DNA in the presence or absence of nucleotide analogues and immunoprecipitated with anti-Scc1 antibodies. Eluted DNA was separated by agarose gel electrophoresis. Input DNA = 5%. Mean ± SEM are shown.

E High temporal resolution kymographs of single Smc1/3 wild-type or KA Scc1$^{\text{Halo-TMR-TEV}}$-cohesin complexes bound to doubly tethered λ-DNA in the presence or absence of ATP and washed with 750 mM NaCl buffer. Scale bar = 5 μm.

Source data are available online for this figure.

 © 2016 The Authors

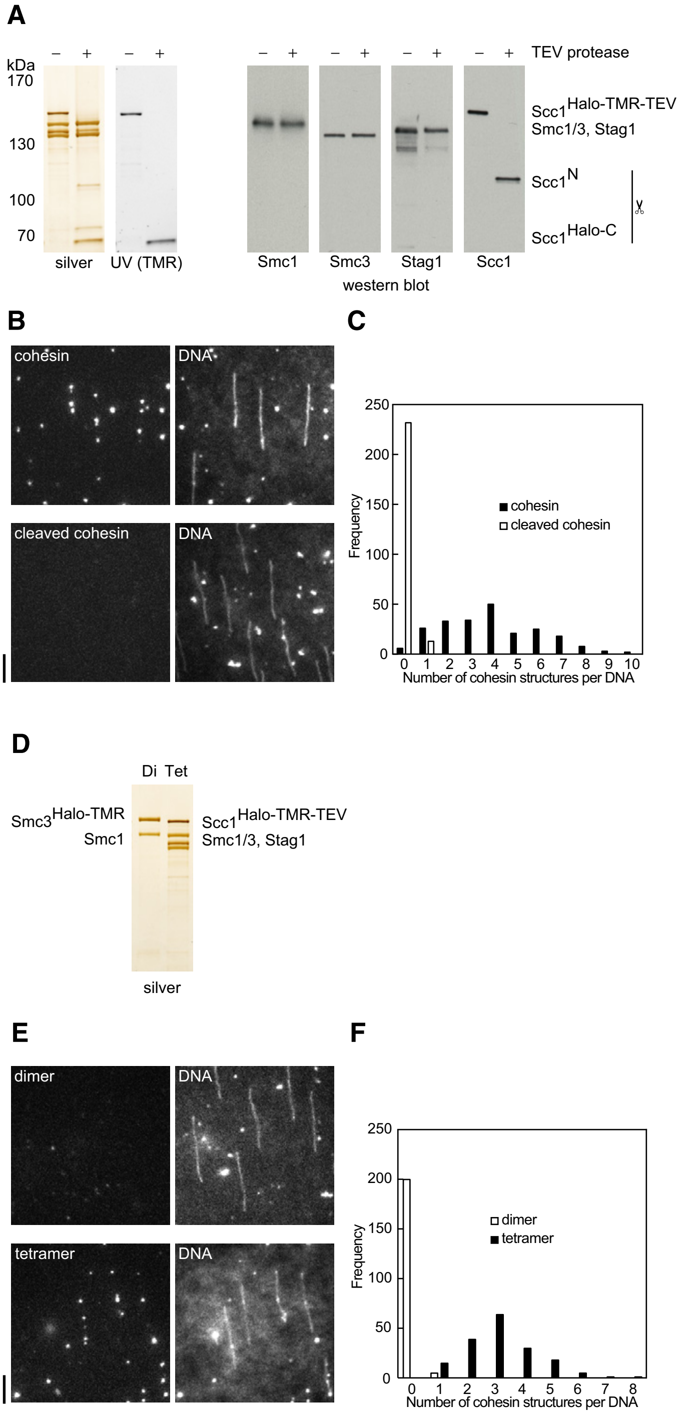

Figure EV3. Cohesin dimers or cleaved tetramers are deficient in DNA binding.

A Silver staining and Western blotting of non-cleaved and cleaved Scc1$^{Halo\text{-}TMR\text{-}TEV}$-cohesin tetramers complexes used in (B, C). TMR was visualized by UV excitation.

B Representative fields of view of λ-DNA flow chambers incubated with non-cleaved (upper panels) or cleaved (lower panels) cohesin and washed with 25 mM NaCl, 25 mM KCl buffer plus Sytox Green.

C Quantification of non-cleaved or cleaved cohesin bound to DNA; 226 DNA molecules were analyzed for non-cleaved, 245 for cleaved.

D Silver stained SDS–polyacrylamide gel of cohesin dimers and tetramers used in (E, F).

E Representative fields of view of λ-DNA flow chambers incubated with cohesin dimers (upper panels) or tetramers (lower panels) and washed with 25 mM NaCl, 25 mM KCl buffer plus Sytox Green.

F Quantification of cohesin dimers or tetramers bound to DNA; 205 DNA molecules were analyzed for dimer, 173 for tetramer.

Data information: Scale bar = 5 μm.
Source data are available online for this figure.

© 2016 The Authors

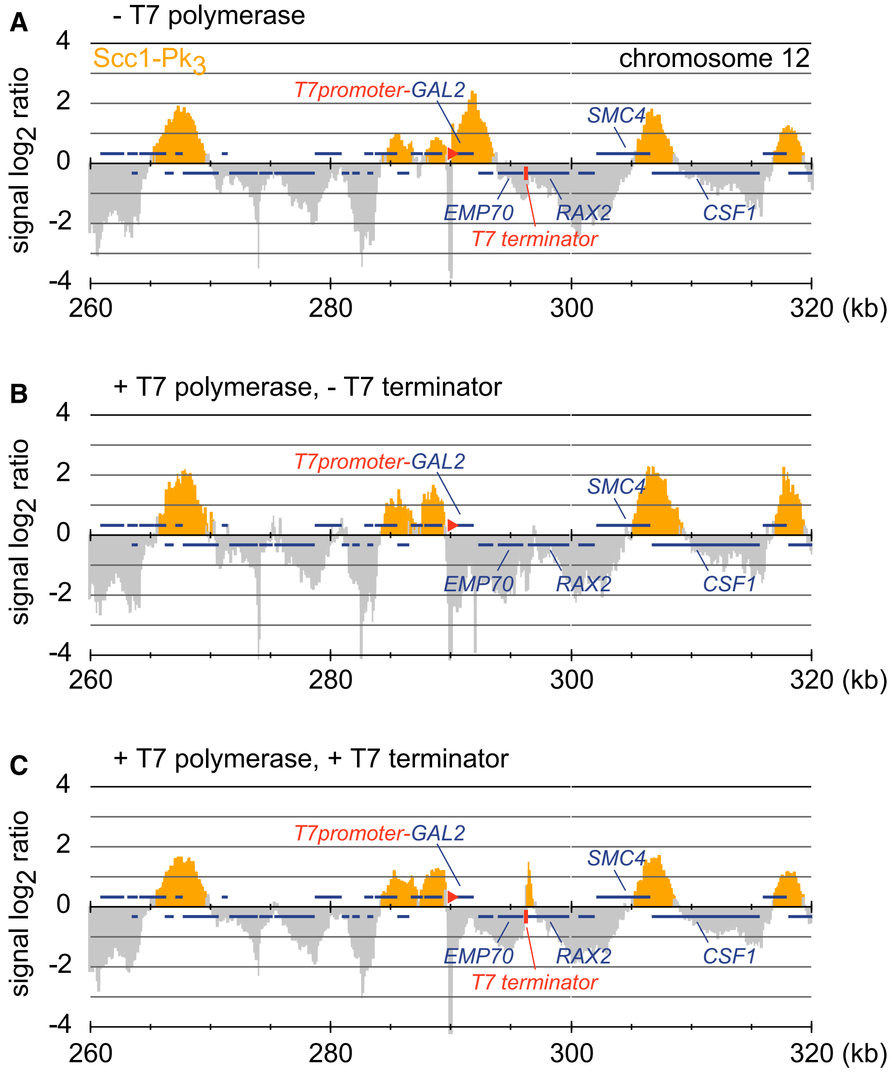

Figure EV4. Cohesin translocation by T7 RNA polymerase transcription in budding yeast.
Cohesin distribution along a section of chromosome 12 is shown, as detected by chromatin immunoprecipitation of the Pk epitope-tagged Scc1 subunit. Yellow coloring indicates a detection *P*-value for the microarray probe set at this location of $P < 0.001$. Blue bars above and below the midline are genes transcribed from left to right and from right to left, respectively. The promoter of the *GAL2* gene was replaced by a T7 promoter consensus sequence, while a T7 terminator sequence was inserted between the *EMP70* and *RAX2* gene.

- A Cohesin distribution in a strain containing the T7 promoter-*GAL2* locus and the T7 terminator, but not expressing T7 RNAP.
- B As in (A), except the strain expresses T7 RNAP fused to a nuclear localization signal under control of the yeast housekeeping *ADH1* promoter, and lacks the T7 terminator sequence.
- C As in (B), but in a strain that includes the T7 terminator sequence.

 © 2016 The Authors
