# Supplementary figures and images for: Rapid movement and transcriptional re‐localization of human cohesin on DNA
Source: EMBO J. 2016 Oct 31;35(24):2671–85. doi: 10.15252/embj.201695402 (PMC5167347; doi:10.15252/embj.201695402)

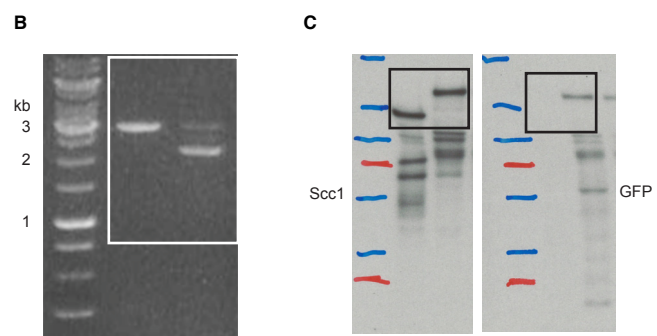

Supplement: Supplementary file 3 — Source Data for Expanded View and Appendix [file EMBJ-35-2671-s003.zip › SourceDataForAppendixFigureS1B-C.pdf]

**A**

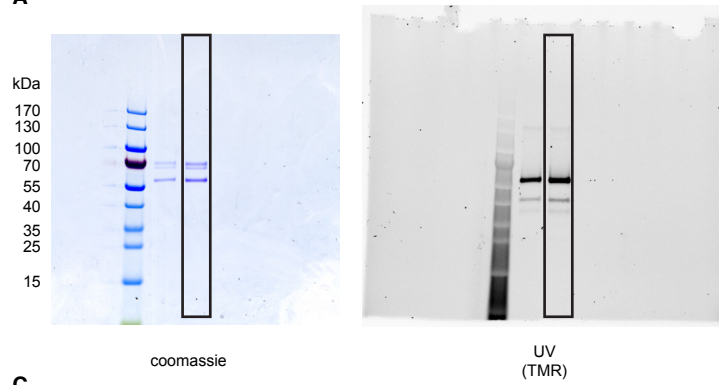

**C**

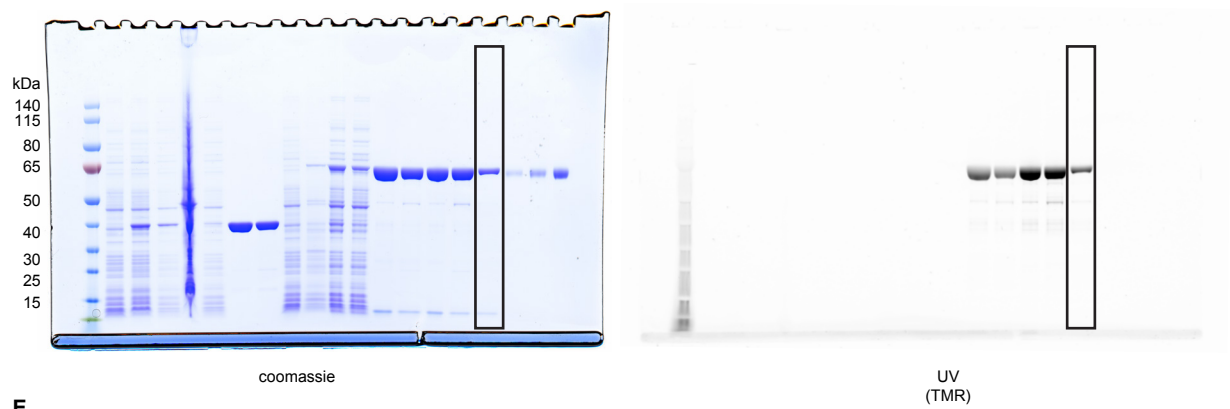

**E**

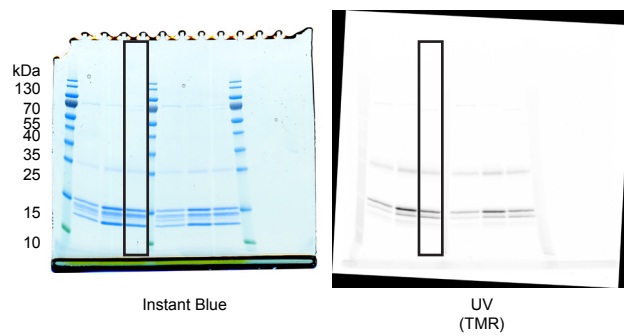

**G**

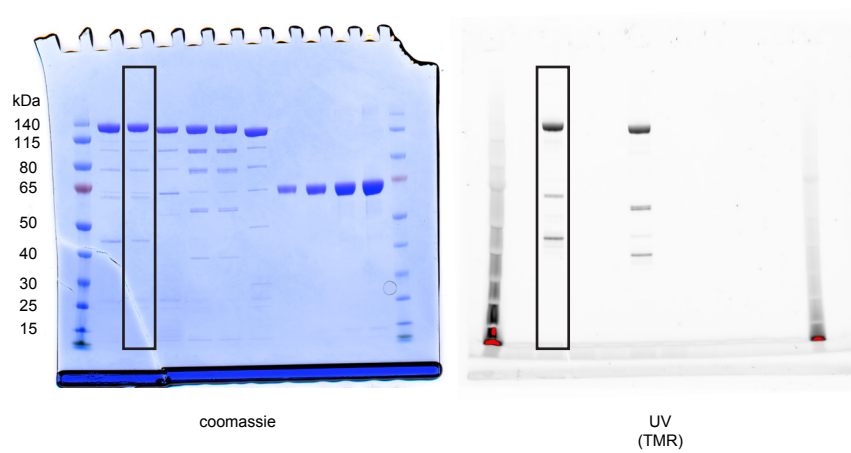

Supplement: Supplementary file 3 — Source Data for Expanded View and Appendix [file EMBJ-35-2671-s003.zip › SourceDataForAppendixFigureS4A,C,E,G.pdf]

**A**

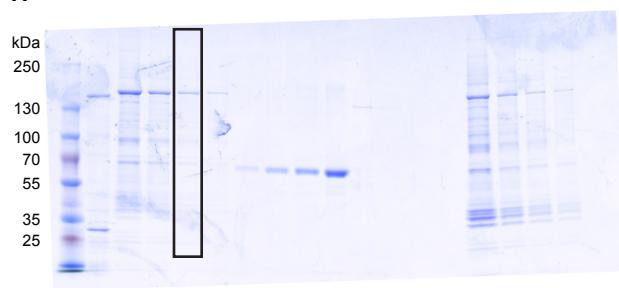

coomassie

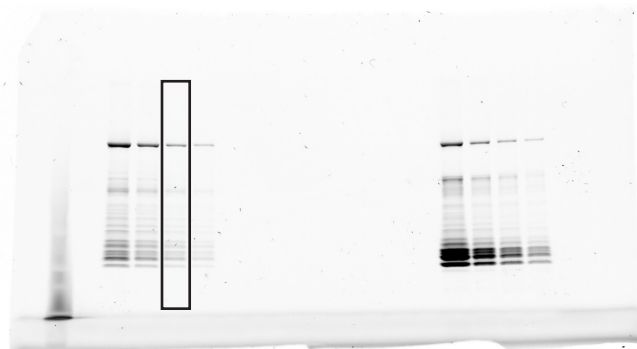

UV (TMR)

**B**

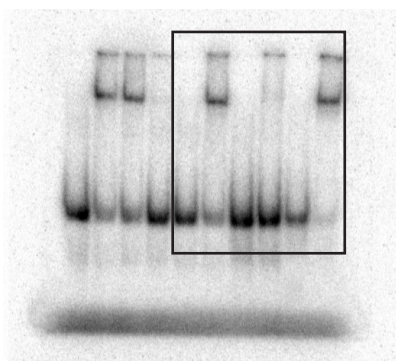

**C**

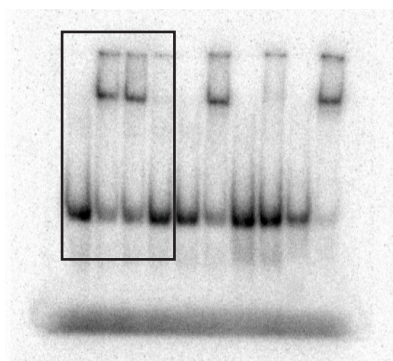

Supplement: Supplementary file 3 — Source Data for Expanded View and Appendix [file EMBJ-35-2671-s003.zip › SourceDataForAppendixFigureS7A-C.pdf]

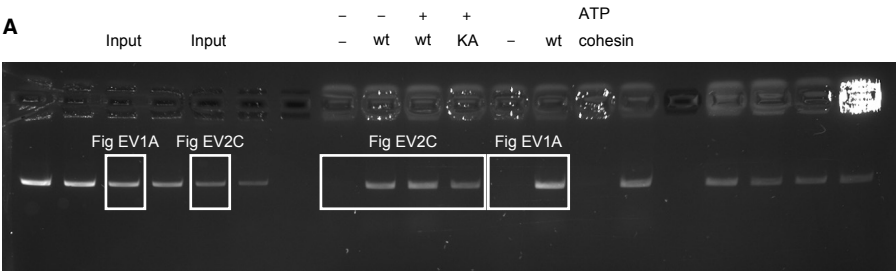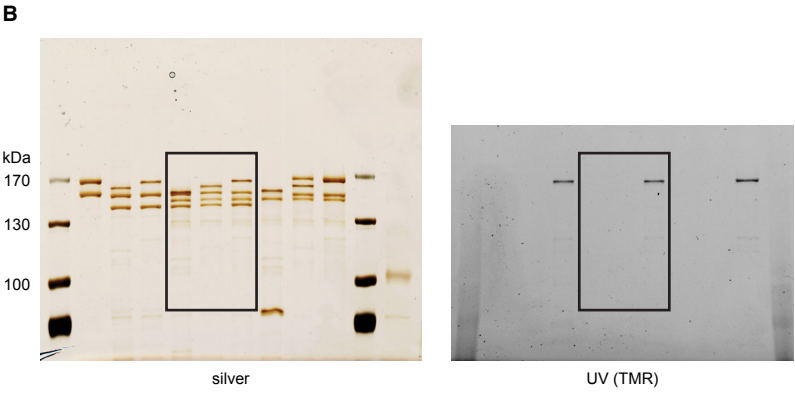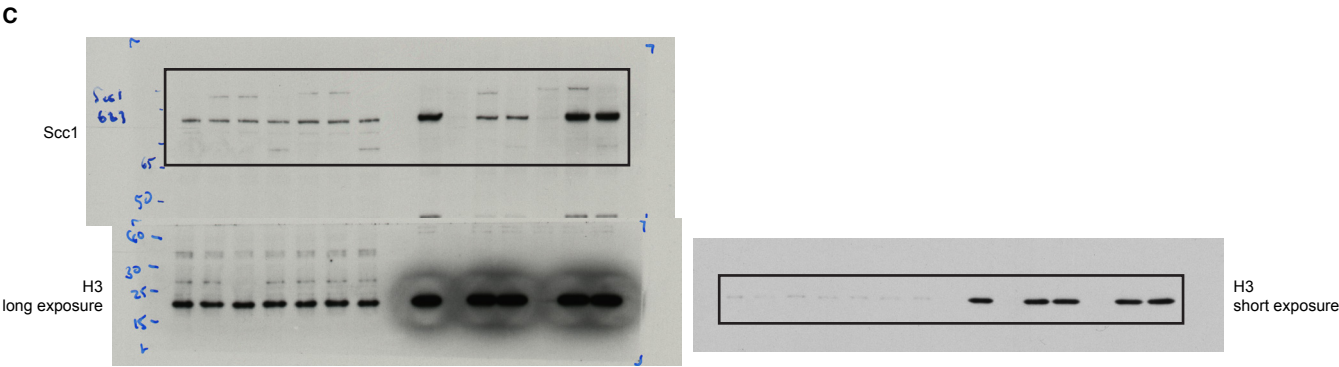

Supplement: Supplementary file 3 — Source Data for Expanded View and Appendix [file EMBJ-35-2671-s003.zip › SourceDataForExpandedViewFigureEV1A-C.pdf]

**A**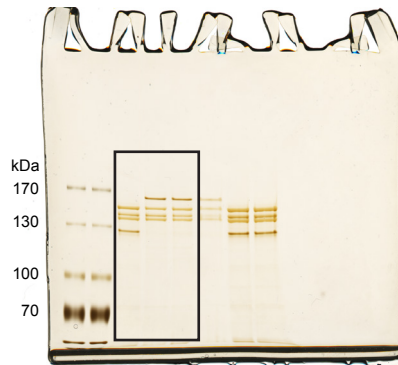**B**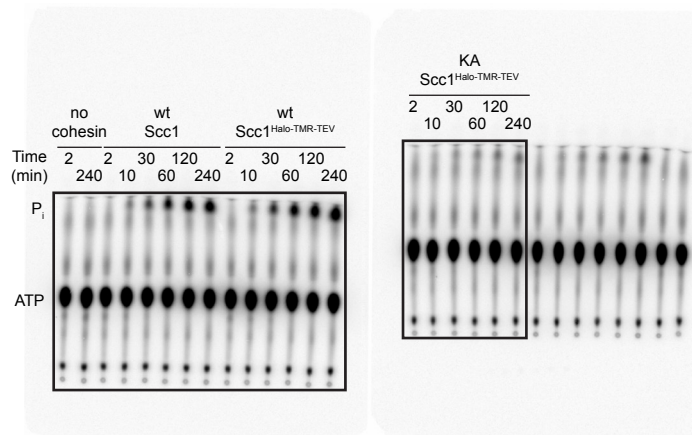**C**

See Source Data for Expanded View Figure 1A

**D**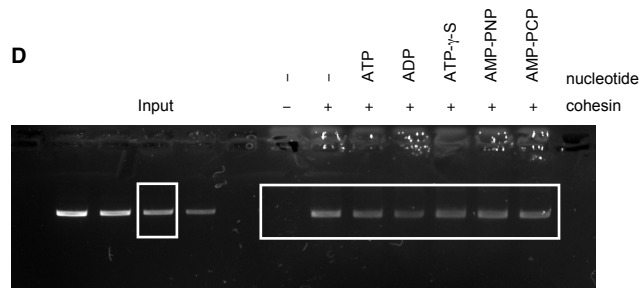

Supplement: Supplementary file 3 — Source Data for Expanded View and Appendix [file EMBJ-35-2671-s003.zip › SourceDataForExpandedViewFigureEV2A-D.pdf]

**A**

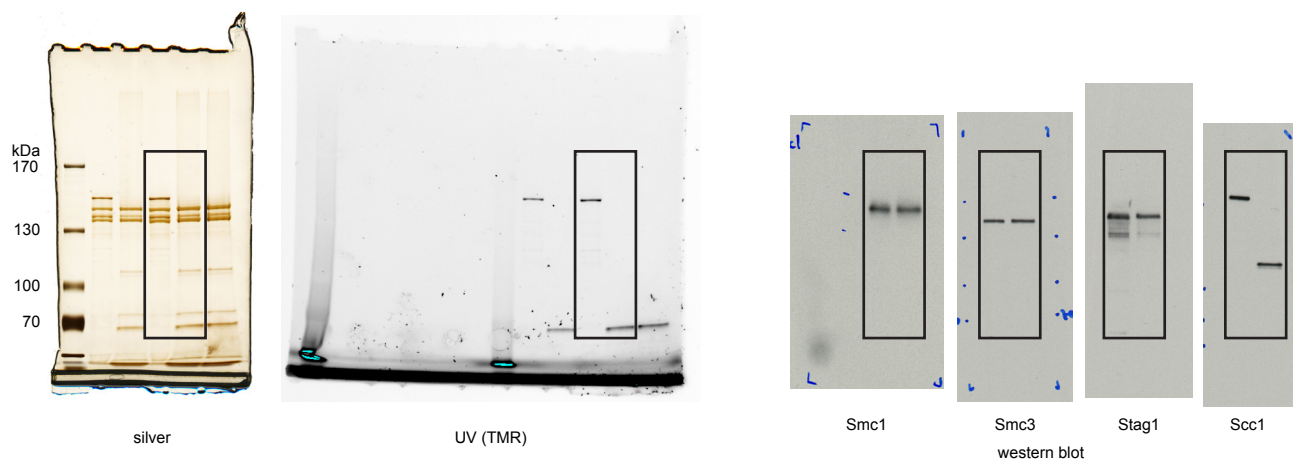

**D**

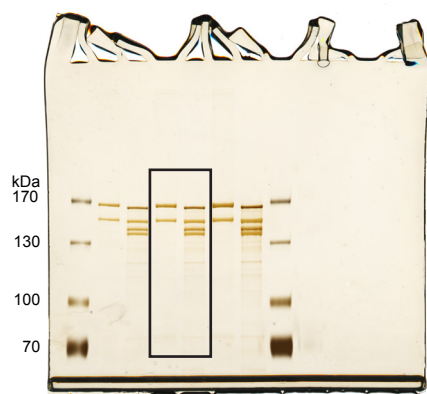

Supplement: Supplementary file 3 — Source Data for Expanded View and Appendix [file EMBJ-35-2671-s003.zip › SourceDataForExpandedViewFigureEV3A,D.pdf]

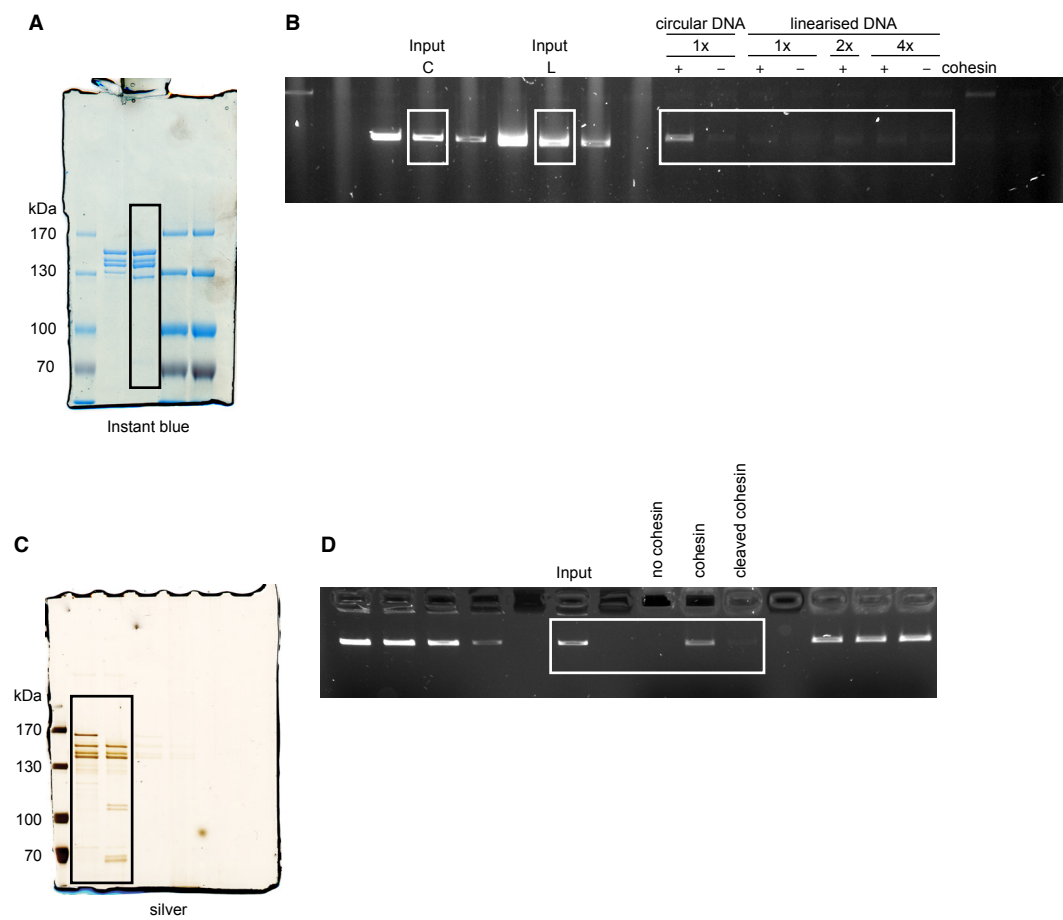

Supplement: Supplementary file 5 — Source Data for Figure 1 [file EMBJ-35-2671-s004.pdf]
